# The psychiatric mental health nurse’s ethical considerations regarding the use of coercive measures – a qualitative interview study

**DOI:** 10.1186/s12912-023-01186-z

**Published:** 2023-01-25

**Authors:** Charlotta Manderius, Kristofer Clintståhl, Karin Sjöström, Karin Örmon

**Affiliations:** 1 Psychiatric assessment unit, adult psychiatry, Region Skane, Helsingborg, Sweden; 2 Psychiatric psychosis unit, adult psychiatry, Region Skane, Helsingborg, Sweden; 3 Department of Care Science, Faculty of Health and Society, Malmö University, Malmö, Sweden; 4 Regionhälsan, The Västra Götaland Competence Centre on Intimate Partner Violence, Gothenburg, Sweden

**Keywords:** Autonomy, Care, Coercive, Considerations, Ethical, Psychiatric mental health nurse

## Abstract

**Background:**

In psychiatric inpatient care, situations arise where it may be necessary to use coercive measures and thereby restrict individual autonomy. The ethical principles of healthcare, i.e., respect for autonomy, beneficence, nonmaleficence, and justice, are recognized as central aspects in healthcare practice, and nurses must be clear about which ethical theories and principles to prioritize and what values are needed for a thorough ethical consideration. The aim of this study is to shed light on psychiatric mental health nurses’ ethical considerations and on the factors influencing them when performing coercive measures.

**Methods:**

This qualitative interview study included twelve psychiatric mental health nurses with experience from psychiatric inpatient care. A content analysis was made. The interviews were audio recorded and transcribed verbatim, and categories were formulated.

**Results:**

The study revealed a duality that created two categories: *Ethical considerations that promote the patient’s autonomy and health* and *Obstacles to ethical considerations*. Based on this duality, ethical considerations were made when performing coercive measures to alleviate suffering and promote health. The result shows a high level of ethical awareness in clinical work. However, a request emerged for more theoretical knowledge about ethical concepts that could be implemented among the staff.

**Conclusion:**

The psychiatric mental health nurses in this study strive to do what is best for the patient, to respect the patient’s autonomy as a guiding principle in all ethical considerations, and to avoid coercive measures. An organizational ethical awareness could increase the understanding of the difficult ethical considerations that nurses face with regard to minimizing the use of coercive measures in the long run.

## Background

According to the International Council of Nurses (ICN) [1], the nurse has an ethical responsibility and must be aware of the power that lies in the practice of the profession. Furthermore, all care has basic humanistic values, according to the Swedish Health and Medical Service Act [2], and this means that people who are cared for according to the Swedish Compulsory Psychiatric Care Act [3] are entitled to the greatest possible autonomy.

Sweden’s municipalities and regions have since 2008 conducted a national improvement work where the goal is to reduce the need for coercive measures in psychiatry, as treating someone against their will and using coercion involves several ethical dilemmas [4]. Furthermore, the Swedish Agency for Health Technology Assessment and Assessment of Social Services (SBU) [4] mentions that preventive measures, other than coercive ones, can to some extent have an effect. Persons cared for under the Swedish Compulsory Psychiatric Care Act [3] are in a vulnerable situation, where the nurse has the important task of protecting the patient from injury as well as providing good care [5].

In a clinical care setting, good care is characterized as individualized, patient focused and related to need; it is provided humanely, through the presence of a caring relationship and by staff who demonstrate involvement, commitment, and concern [6]. It differs somewhat from beneficence, as proposed by Beauchamp and Childress [7], which refers to acts of kindness, charity, and altruism, where a beneficent person does more than the bare minimum.

With this in mind, it can be problematic for the nurses to engage in ethical considerations that are in favor of or against a coercive measure, according to Olofsson et al. [8]. Refraining from a coercive measure can be just as devastating as carrying it out in certain situations. Thus, failure to carry out a coercive measure may go against doing the right thing, but on the other hand, the coercive measure may infringe the patient’s integrity, autonomy, and dignity.

In Sweden, the conditions for applying the Compulsory Psychiatric Care Act [3] are as follows: the person suffers from a serious mental disorder; the person has an indispensable need for psychiatric care, which cannot be met in any other way than through qualified psychiatric round-the-clock care; the person opposes care or, because of their mental condition, lacks the ability to take a stand on the issue. Compulsory care may not be provided if the patient’s mental disorder consists only of an intellectual disability. Something that must also be considered is whether the patient, because of their mental disorder, is dangerous to another’s personal safety or physical or mental health. Medical restraints, like fixation, forced medical treatment, such as injections of medication and seclusions, are some coercive measures that are relevant to use during the application of the Compulsory Psychiatric Care Act [3]. These three coercive measures may create ethically difficult situations for the nurse due to the risks that may arise in connection with the measures [9].

There are also, according to Szmukler and Appelbaum, several kinds of informal coercion or treatment pressures, such as persuasion, interpersonal leverage, inducements, and threats before the use of compulsory treatment [10], that may be ethically challenging for nurses.

Coercive measures are not only integrity challenging but are also related to serious risks for mentally ill patients. Trauma similar to posttraumatic stress disorder has been reported [11], as well as suicidal attempts and self-damage [12]. Other complications are cardiac arrest and pulmonary thrombo-embolism, which could be fatal, especially in connection to longer periods of restraint [13, 14]. Moreover, according to a Cochrane review, there is no evidence that coercive care, such as seclusion and restraint, benefits mental health [15].

Coercive measures may also create ethically difficult situations for the nurse due to the risks that may arise in connection with the measures [1]. There is, for instance, a risk that patients will not be able to communicate their wishes [16], which may lead to violation of the patient’s dignity [17] and, in turn, to a deteriorating treatment alliance [5]. Ethical dilemmas may also occur when there are different perceptions of what is right and wrong in treatment, as everyone has their own perceptions of and values regarding what is good and bad [18, 19]. According to Andersson et al. [18], coercive measures, such as mechanical restraint, are an established part of care where restraints are an act of good will and considered necessary to protect the patients from injuring themselves, although the nurse desires to provide care and relieve suffering. It is important to keep in mind that all care must be provided with respect for the equal value of people and for the dignity of the individual, according to the Health and Medical Services Act [2].

The psychiatric mental health nurse must work to maintain respect for the person’s dignity, integrity, and self-determination; give the individual the opportunity to experience trust, meaning, and hope; work to support other employees in achieving a higher ethical awareness; and be aware of when respect for fundamental values is threatened [1]. Olofsson et al. [8] have highlighted the importance of protecting the patient’s health from employees’ wrongdoing. By reflecting on and being responsible for ethics in the workplace, the nurses can challenge their own ethical competence [1] and have a responsibility to give life to the ethical discussions to strengthen the quality of nursing by developing an ethical compass among the staff.

Being able to reflect ethically, having ethical knowledge, acting ethically, behaving ethically, and engaging in ethical considerations, are the basis for all nursing care. Previous studies have described the nurse’s experience of performing coercive measures [5, 8, 18, 20]. However, few studies have explained what ethical considerations nurses engage in when performing coercive measures [21, 22]. Beauchamp and Childress [7] have presented a framework for the ethical assessment of alternative courses of action in healthcare, where the following four principles are particularly central: respecting patient autonomy, acting on the principle of beneficence, being aware of the principle of no harm, and attending to the principle of justice. These four principles may be a starting point for ethical considerations. Other principles, such as trust, care, and solidarity, are of no less value, but the nurse must decide what promotes health and relieves suffering. According to Hem et al. [21], there is, at present, a growing awareness of ethical challenges. Hence, modern healthcare values the patient’s autonomy higher than in the past. It is statutory that care should be designed and implemented in consultation with the patient as far as possible [2]. One of the challenges for the nurse is to balance the patient’s autonomy with the safety of others and at the same time let the patient participate without coercion [21]. The authors of this study believe that nurses must, through careful ethical consideration, clarify for themselves which ethical theories and principles are of priority and what values are achieved in each specific situation. In psychiatric practice, the psychiatric mental health nurse faces ethical considerations daily about what should be done and what is considered the right thing to do for the patient, which in turn may create conflicts of conscience. In a study by Jensen and Lidell [23], nurses believed that their own conscience played a major role in ethical considerations and that it was important to stand up for their own ethical values and listen to their conscience. Several different factors, such as previous experiences, interpersonal and collegial relationships, emotions, laws, rules, and constellations of power, influence the psychiatric mental health nurse’s ethical considerations. In particular, it is ethically difficult when the nurse has to implement coercive measures without being involved in the decision [5, 8]. Even so, according to the ICN [1], the psychiatric mental health nurse has a responsibility to lead the ethical discussion in order to strengthen the quality of nursing. Therefore, the aim of this study is to further explore this research area with the purpose of examining the psychiatric mental health nurse’s ethical considerations and investigating what factors are of importance for ethical considerations when using coercive measures.

## Methods

### Design, sample, and setting

The study was designed as a qualitative interview study. The aim was a selection of participants that would lead to an increased understanding of variations within the subject to be studied. The inclusion criteria were psychiatric mental health nurses, with a one-year master exam, who worked or had recently worked in inpatient psychiatric care and participated in and ethically reflected on coercive measures. The study was conducted in the southern part of Sweden. The recruitment of participants was done in 2021, during the month of February. Men and women of different ages and with experience from different psychiatric clinics, were included, in order to get the greatest possible variation in the answers (Table 1).

**Table 1:** Demographic data of participants (*N* = 12)

Gender	
Men n (%)	2 (17)
Women n (%)	10 (83)
Age (year) Mdn	45
Work experience **(**year) Mdn	
Nurse (Rn)	13
Psychiatric mental health nurse (PRN)	10

### Data collection

Semi-structured [24] interview questions were developed through conversations and reflections among the authors. The questions aimed to capture the psychiatric mental health nurse’s ethical considerations when performing coercive measures, without intent to study any particular coercive measures. A total of twelve psychiatric mental health nurses were interviewed. The authors chose to conduct a preparatory interview to investigate whether the questions asked were relevant and clear for the purpose of the study. During the preparatory interview, it emerged that the participant would have preferred to see the questions in advance as the subject required reflection. Following the preparatory interview, the other participants therefore received sample questions together with information about the purpose of the study and a consent form. After the preparatory interview, the questions were slightly adjusted for clarification without altering their essence. Hence, the preparatory interview was included in the results. Nine of the participants chose to be interviewed at their workplace and three to be interviewed through the digital conference Zoom. The duration of the interviews was between 30 and 60 minutes, and they were recorded and transcribed verbatim.

### Data analysis

Collected data were analyzed by the authors, according to Burnard [25], who describes fourteen steps to perform a manifest qualitative content analysis. Twelve of the fourteen steps were used in the analysis. The category check by respondents was excluded as well as comparing the collected data with previous research. The transcript was read repeatedly in order to delve into the material and describe all aspects of the content. An open coding was done by giving each emerging topic a heading, and the headings were merged into higher-order headings. The procedure was repeated to further shape the central parts of the interview. The transcripts were re-examined and compared with the categories obtained so that the latter covered all parts of the content of the transcripts. Data that corresponded to the purpose of the study were coded into different sections. Each coded section of the interviews was then cut out of the transcript and all parts of each code were collected. The cut-out sections were pasted into a separate document with appropriate headings and subheadings. The findings were collected under each respective heading and category. In order to ensure that the findings were not taken out of context, both a complete transcript of the interview and the original audio recording were always available. A compilation of the results was started by processing each section separately, and representative quotations were selected. The authors remained open to the interview material throughout the writing process (see Table 2, for the process of analysis).

**Table 2:** Process of analysis according to Burnard (1991)

Transcript	Open coding	Heading	Category
Coercive measures are for the patient’s best to alleviate various difficult [mental] conditions… it is difficult for the patient to be in a manic state, for example, and sometimes we have to do coercive measures like ECT, but patients may thank us afterwards because it is about relieving suffering.	Coercive measures are in the patient’s best interests to alleviate severe [mental] conditions.	Ethical considerations to reduce suffering and for the benefit of the patient	Ethical considerations promoting patient autonomy and health

### Trustworthiness

The authors aimed for trustworthiness in accordance with standard criteria for qualitative research [26]. The thorough description of design and method, as well as the description of setting and participants and the choice of quotations to strengthen the result, enriched the credibility of the results. The authors discussed the material throughout the progression of the analysis, which also enhanced the credibility. Two of the authors conducted the interviews, as well as using an interview guide, which was beneficial for achieving dependability.

Three of the authors are psychiatric mental health nurses and one of the authors is a psychiatrist and psychotherapist. All authors are experienced in psychiatric in- and outpatient care and in performing coercive measures. The fact that the authors have experiences from a variety of psychiatric care contexts has been valuable throughout the methodological process.

### Ethical research considerations

This study was based on four ethical research principles: the information requirement, the consent requirement, the confidentiality requirement, and the utilization requirement [27]. Prior to the interview, the participants received written information that the interviews would be recorded, that the material would be de-identified, that it was voluntary to participate, and that they could withdraw at any time**.** Written informed consent was obtained from all participants prior to the interview. The study was approved by the university and the psychiatric department of the hospital. Approval from an Ethics Committee was not required for this type of study as it could not be traced to individual participants, did not process sensitive personal data according to the General Data Protection Regulation (GDPR) [28], and did not affect the participants physically or mentally [29].

## Results

This study found a duality between the psychiatric mental health nurses’ ethical considerations with regard to coercive measures and the obstacles to those considerations. This finding led to the creation of two categories and five headings, in accordance with Burnard [25] (see Table 3).

**Table 3:** Category system

Headings	Categories
Ethical considerations to reduce suffering and for the benefit of the patient	Ethical considerations promoting patient autonomy and health
Ethical considerations based on personal ethical principles	
Ethical considerations create relationships and trust	
Loneliness in the profession	Obstacles to ethical considerations
Lack of understanding of the psychiatric mental health nurse’s ethical dilemmas	

It emerged that the psychiatric mental health nurses in this study saw coercive measures as major threats to the patient’s autonomy. At the same time, they all considered coercive measures to be necessary on certain occasions, in order to alleviate suffering and promote patient health. It also emerged that ethical considerations were hindered by professional loneliness and a lack of understanding on the part of other staff. It was in this predicament that that the psychiatric mental health nurses engaged in ethical considerations when performing coercive measures.

*“We are not allowed to exercise power over the patient and coercive measures are the last measure we should resort to.” (Participant 6).*

### Ethical considerations promoting patient autonomy and health

This category describes ethical considerations made by the psychiatric mental health nurses in order to preserve the patient’s autonomy in each situation where coercive measures were considered. The ethical considerations, which were also based on personal ethical principles, were made for the benefit of the patient, in order to prevent coercive measures as far as possible.

#### Ethical considerations to reduce suffering for the benefit of the patient

A number of ethical considerations were made with the patient’s best interests in mind. There was a desire to preserve the patient’s autonomy and dignity, by involving the patient as much as possible in the decisions, and to persuade the patient to receive help on a voluntary basis. The participants were clearly aware that the patient’s condition could be improved in both the short and the long run through coercive measures. As the patient’s suffering was reduced, ethical considerations were facilitated.

*“Coercive measures are used for the benefit of the patient and to break various difficult conditions. … It’s hard, but the patients may thank us afterwards because it’s about alleviating suffering.” (Participant 3).*

The participants also believed that it was in the patients’ best interests to prevent them from forcibly causing harm to fellow patients and themselves, which later could lead to an increase in suffering. The importance of nursing knowledge and of measures being based on the ethical principle of beneficence, was expressed by all participants. Furthermore, the participants agreed on those ethical considerations being situational, always based on the patients’ current mood, and requiring attention to whether it was necessary or not to carry out a coercive measure. The preservation of dignity was seen as an important principle in nursing.

*“Preserve the patient’s dignity. That is also a consideration. Not only here and now but in the long term.” (Participant 12).*

In their ethical considerations, participants thought a lot about the patient’s vulnerability. Not exposing the patient’s body more than necessary when giving forced injections, or only allowing female staff to be present in a coercive procedure if the patient had previously been mistreated by men, were examples of situations where the patient’s vulnerability was considered. The participants also stated that seclusion could sometimes be justified in order to preserve patient dignity. The ethical consideration in that situation was about protecting both the patient in question and other patients from embarrassment or shameful suffering. All participants emphasized that coercive measures should never be a punishment and that non-urgent coercive measures called for careful planning in order to maintain the patient’s autonomy and dignity, and at the same time minimize suffering. The participants stated that forced injections could be traumatic, but by informing and involving the patient, the trauma would be as minimal as possible. The patients’ participation could be promoted by allowing them to choose the method of administration of the drug, the place of administration, the position, on which side the injection would be given, who would perform the procedure, how many would be in the room, and who would remain with the patient afterwards. It also emerged that participation could be increased by giving patients the opportunity to tell how they felt afterwards. The ethical consideration was about promoting the patient’s self-determination through participation in order to minimize any feeling of abuse of autonomy.

*“The important thing is to make the patient understand what we’re doing, for then the patient may experience the coercive measure as less of a violation, I believe.” (Participant 6).*

None of the participants considered it unproblematic to carry out coercive measures, but those performed were considered ethically defensible as the patient’s wellbeing was in focus. The ethical considerations revolved around having to oppose the patient’s autonomy while promoting the patient’s health and, hence, reducing suffering in the end.

#### Ethical considerations based on personal ethical principles

The nurses’ own ethical point of view was important for their ethical considerations and the differences in their ethical reasoning that emerged were related to experience and self-esteem. Many participants stated that they maintained internal ethical monologues that concerned their conscience as well as loyalty and solidarity towards patients and staff.

*“We nurses must be more assertive. Talk and listen to the patient. Not prescribe a coercive measure and then listen, but listen and then prescribe, if it so happens that it’s necessary.” (Participant 6).*

According to the participants, it felt more right to act on their own conscience than on the mere duty to implement a coercive measure that they felt could not be justified, especially if the purpose of the coercive measure did not correspond to a fair and humane treatment. The majority expressed that experience contributed to both thoughtfulness and more carefully thought-out and planned ethical considerations. Some expressed that earlier in their professional careers, when less experienced, they valued duty higher than their own ethical convictions, in, for example, enforcing a doctor’s compulsory prescriptions even though the participants did not consider it fair or in the patient’s best interests. When less experienced, the participants relied more on the skills of others than on their own skills.

*“The ethical aspect has become more difficult because you sort of think more about the situation today than you did earlier. Before, it was more a matter of following the prescription given by the doctor, it was my duty.” (Participant 8).*

Some coercive measures, such as involuntary injections, were more difficult for the participants to handle. The ethical considerations were along the lines that it hurt for a short while but was beneficial for the patient in the long run. One of the participants stated that force-feeding was an example of the patient’s health being promoted in the long term, despite increased patient anxiety both during and after the coercive measure. Another difficult ethical dilemma arose when patients suffered from cognitive impairment or dementia and the nurses were not able to communicate with the patient or convey the purpose of the coercive measure. According to several participants, it was also ethically challenging when patients perceived that they did not have a choice.

“*The thing about ethical considerations, how to draw the line for a coercive measure. […] my conviction [was] that I should do everything I could to make the patient take the pill in order to avoid it [the injection] and not just do it automatically.*” (Participant 1).

Even if the patient agreed to receive a forced medical injection, the participants sometimes perceived that the patient’s autonomy was violated, which was seen as a dilemma. It emerged, however, that for the participants, treatment pressures felt better for their ethical conscience than if the patient risked being subjected to stronger coercive measures, such as mechanical restraint, to obtain the prescribed injection. When it was necessary to ask for assistance from other departments for a planned coercive measure, the nurses felt that the increased number of staff could lead to a feeling of inferiority in the patient, a feeling that might subsequently be consolidated. In such cases, preventing the exercise of power and the exploitation of the patient’s vulnerability and weakness was uppermost in the participant’s ethical considerations. Informal coercion was another problematic ethical issue, however, and it was also considered problematic to document it in a proper way.

#### Ethical considerations create relationships and trust

The participants were aware that the relationship with the patient sometimes varied depending on the medical condition. Many participants emphasized that preventive measures were crucial in creating trust and a relationship with the patient, in order to avoid having to apply coercive measures. The foundation for nursing interventions was considered to be accurate treatment based on good communication.

*“All these things that we do are very much built on relations. The patient should know that I do all this because the patient needs this care and not because I want to use my power.” (Participant 7).*

According to most participants, there was a possibility that the relationship between patient and nurse would be influenced in the execution of coercive measures, which in turn affected the ethical considerations. The ethical considerations revolved around the fact that the relationship risked being temporarily damaged at the same time as the coercive measure improved the patient’s mood. The majority of the participants reflected on whether the person who had the best relationship with the patient was the one who should carry out the coercive measure or not. In most cases, such decisions were made based on the situation and the existing relationship. It was suggested that the patient could be involved by being able to choose who would perform a coercive measure, which would avoid damaging an existing relationship.

*“Give the patient a choice, involve the patient.” (Participant 9).*

The majority of the participants stated that it was particularly difficult to maintain a relationship with patients who lacked insight into their mental illness. The relationship was often affected by the current state of the patient’s illness, such as manic relapses, psychotic breakthroughs, and paranoia. Dementia diseases, cognitive impairment, language confusion, and language barriers were perceived by the participants as an obstacle to relationship building. Not being able to communicate was difficult, which, in turn, made it difficult to create trust and thus build a relationship. The relationship between patient and nurse was important in the preventive work to avoid coercive measures.

“*[The patient] was completely convinced that this situation would lead to their death. This was difficult to meet … it was both the language barrier and this conviction that the patient had that we were actually in a concentration camp.*” (Participant 1).

Many of the participants expressed that it felt difficult not to be able to convey the purpose of the coercive measure to the patient. Lack of communication could create a feeling of abuse and thus increase the patient’s suffering. The ethical considerations in these situations were particularly difficult for the psychiatric mental health nurses.

### Obstacles to ethical considerations

In this category, it emerged that obstructive factors affected the participants while they were making ethical considerations, thereby hindering the preservation of the patient’s autonomy. The obstructive factors in question were loneliness in the profession and a lack of understanding of the ethical dilemma.

#### Loneliness in the profession

It emerged during the interviews that the participants felt that a high level of ethical awareness was required in order to provide psychiatric care. It also emerged that the participants felt that there were shortcomings in the understanding of the psychiatric mental health nurses’ ethical considerations, which tended to complicate the nursing work in connection to coercive measures. The participants stated that they sometimes experienced pressure for coercive measures from other categories of staff and that this contributed to a feeling of loneliness in their ethical considerations.

“*If you don’t have [ethical] knowledge, then things can go so wrong when coercive measures are applied. They mean well but do the wrong thing. It’s often a matter of lacking knowledge. So, education is in fact the foundation for everything.*” (Participant 8).

The majority of the participants requested more ethical knowledge, which they believed would enable a better response and a greater understanding of the patient’s current illness. Several participants claimed that they had received pressure from nursing assistants regarding coercive measures and medication before other nursing measures had been evaluated. Some of the participants also stated that they felt questioned in their competence and profession, which constituted an obstacle to their ethical considerations. It emerged that not only pressure from nursing assistants but also disrespectful treatment from doctors led to some of the psychiatric mental health nurses feeling lonely in their profession and in their ethical assessments.

“*I’ve been pressured by nursing assistants who think that if a patient becomes aggressive and threatening, then it’s mechanical restraints and injections that should be resorted to; there aren’t a lot of alternatives, sort of. And then I think that it has a lot to do with ignorance.*” (Participant 9).

Some participants gave examples of how doctors sometimes showed a lack of respect in coercive situations by giving the injection to the patient when the nurse had made a different assessment and did not consider the measure ethically justified. Another participant stated that a doctor had threatened to report if the coercive measure was not carried out as prescribed. A third participant stated that doctors occasionally threatened uncompromising patients with the use of belts even though there was no indication for such a measure.

“*Sometimes on-call doctors enter and prescribe before they have listened, they have heard us but not listened. Some of them have already made their minds up when they hear the patient’s name. Taking their cue from earlier experiences [of the patient]*.” (Participant 6).

The majority of the participants claimed that due to a lack of staff, the staff could not always perceive shifts in the patient’s mood and were thus unable to divert and calm the patient in time. In these situations, there was an increased risk of coercive measures, and the psychiatric mental health nurses’ ethical considerations were about protecting patients from unfair treatment and unnecessary suffering caused by staff shortages.

#### Lack of understanding of the psychiatric mental health nurse’s ethical dilemmas

It emerged during the interviews that there was a need for an increase in ethical competence in the organization, as many of the participants were alone in their ethical considerations regarding coercive measures. The participants felt that by raising the staff’s ethical competence, the understanding of the psychiatric mental health nurses’ profession and of their ethical considerations could increase. The majority of the participants also felt that more experienced nurses were needed. The less experienced psychiatric mental health nurses were affected to a greater extent by the other nurses’ opinions rather than trusting their own assessments.

“*It would have been good with more nurses. Nursing assistants who have worked for many years may easily influence new nurses … and the new [nurses] maybe don’t really dare be assertive about themselves and their judgements*.” (Participant 9).

Several participants stated that there was an ongoing ethical discussion among nurses in the workplace, but that, although there was time for reflection, not all staff members participated. On the other hand, many participants asserted that they had support from colleagues and opportunity for discussion in the work team after a troublesome coercive situation. Nevertheless, many of the participants requested an ethical discussion forum organized by the employer. A majority believed that ethics rounds would contribute to a higher ethical awareness, which in turn could lead to a better response to and a greater understanding of the patient’s situation as well as of the psychiatric mental health nurses’ ethical dilemmas.

“*But we nurses talk a lot about it [ethics], about being hospitalized in psychiatric inpatient care, [about how] that very fact means that there should be great acceptance of their not behaving like people in the street*.” (Participant 10).

Some participants wished for a greater ethical commitment on the part of the employer, but at the same time they felt that this was not prioritized.

## Discussion

All participants stated that ethical considerations, based on ethical principles and on the desire to preserve the patient’s autonomy as far as possible, were applied in their work with patients. The results showed that the psychiatric mental health nurses did everything to increase the patient’s participation and autonomy. It emerged that when the purpose of a particular measure could not be communicated, this led to violation of the patient’s autonomy and a decrease in patient participation. Previous studies [30, 31] emphasize the importance of good communication in order to build a genuine and meaningful relationship between patient and nurse. Results from the present study showed that patient participation was highly valued in order to maintain and increase patient autonomy, and that the psychiatric mental health nurses strived to avoid coercive measures.

The aforementioned desire to preserve the patient’s autonomy and dignity by involving the patient as much as possible in the decisions regarding their care, could be a reason to introduce the question regarding autonomy and coercive measures already in the care plan for outpatients or at admission. This is highlighted in a review by Chieze et al. [32], where it is suggested that by discussing coercive measures with the patient and involving the patient in the care planning phase, the patient may agree with the caregiver that coercion is the best way to overcome a mental crisis. By adhering to such a procedure, coercion can be seen as a way to enhance the patient’s condition in certain circumstances, which is in line with the view expressed by the participants in the present study, namely, that coercion can sometimes be used to decrease suffering both short and long term.

Lack of ethical awareness negatively affected ethical considerations and therefore counteracted the patient’s autonomy. Inadequate ethical competence among colleagues led to ethical conflicts and increased moral stress, as also shown in a study by Pauly et al. [33]. According to Eren [34], negative attitudes may influence the psychiatric mental health nurse’s relationship with the patient, and a deteriorating relationship may lead to restrictions on patient autonomy. Another conclusion in Eren’s [34] study was that nurses in psychiatric care needed further ethical education. The results of the present study showed a desire to increase ethical competence within the entire care organization, so that everyone in the team would become more involved and gain understanding of the psychiatric mental health nurses’ ethical dilemmas and considerations. As demonstrated in a review by Paradis-Gagné et al. [35], decisions regarding coercive measures should be made in careful consultation with team members, while minimizing the restriction of patient autonomy. Furthermore, previous studies [35, 36] showed that a common ethical value base within the care team is crucial for fulfilling the patient’s wishes for a genuine meeting between patient and staff and for the promotion of the patient’s autonomy. This is in line with our study participants’ view that ethics rounds and an ethical commitment on the part of the organization management would be of great value. Ethics rounds may foster cooperation among the staff and make them learn to see things from different perspectives, which in turn may influence patient care [37]. In conclusion, the integration and application of ethical awareness in the mental health care organization and of ethical values among mental health staff may be beneficial for patients’ autonomy and participation when coercive measures are needed.

### Clinical implications

In this study, patient participation was highly valued by all psychiatric mental health nurses, in order to maintain as well as increasing patient autonomy. One way to increase the patient’s autonomy could be to discuss a possible coercive measure already in the care plan. Even if the psychiatric mental health nurses always strive to avoid coercive measures, the patient may, in certain circumstances, agree with the caregiver that coercion is the best way to overcome a crisis. Another clinical suggestion is to introduce ethics rounds in addition to medical rounds. According to a majority of the study participants, ethics rounds could improve ethical awareness among the staff and thereby also improve the staff’s response to and understanding of both the patients’ situation and the psychiatric mental health nurses’ ethical dilemmas.

### Limitations

Graneheim et al. [38] highlight the challenge of using qualitative content analysis in research. Maintaining a “common thread” throughout the study, as well as enabling the reader to distinguish the voice of the researcher from that of the participants, is necessary for establishing rigor [38]. Therefore, it was important for the researchers in this study to thoroughly describe design and method, as well as setting and participants, and to use quotations from the interviews. One limitation of the study may be the number of participants, and a sample of twelve may be considered small. However, according to Malterud et al. (2016), the more information a sample holds, the lower the required number of participants [39]. Another limitation could be that the participants worked in a limited geographical region, which could affect the result since working methods, education, and ethical awareness may vary between regions. However, data from qualitative interview studies are not aiming for generalizability.

## Conclusion

We found strong agreement that all psychiatric mental health nurses in our study actively consider ethical dilemmas connected to coercive measures. Coercive measures were only used in exceptional circumstances and the respect for the patient’s autonomy was prominent in the ethical reasoning of all the psychiatric mental health nurses. The four ethical principles, involving respect for autonomy, beneficence, nonmaleficence, and justice, were constantly present in their ethical considerations. Coercive measures were seen as justified in both a short- and a long-term perspective to alleviate patients’ suffering. Obstacles to ethical considerations were professional loneliness and lack of understanding on the part of other staff members about the psychiatric mental health nurses’ ethical dilemmas. Theoretical knowledge about ethical concepts was asked for.

## Data Availability

The datasets generated and/or analyzed during the current study are available from the corresponding author on reasonable request.

## References

[1] International Council of Nurses (2022). Code of ethics [internet].

[2] Hälso- och sjukvårdslagen [The Health and Medical Services Act] (SFS 2017:30) [Internet]. Stockholm: Socialdepartementet [The Ministry of Health and Social Affairs]; 2017. Available from: https://www.riksdagen.se/sv/dokument-lagar/dokument/svensk-forfattningssamling/halso%2D%2Doch-sjukvardslag-201730_sfs-2017-30. Accessed 27 Jan 2022.

[3] Lagen om psykiatrisk tvångsvård, 1991:1128 [The Swedish Compulsory Psychiatric Care Act]. [Internet]. Stockholm: Socialdepartementet [The Ministry of Health and Social Affairs]; 1991. Available from: https://www.riksdagen.se/sv/dokument-lagar/dokument/svensk-forfattningssamling/lag-19911128-om-psykiatrisk-tvangsvard_sfs-1991-1128. Accessed 27 Jan 2022.

[4] SBU [The Swedish Agency for Health Technology Assessment and Assessment of Social Services]. (2019) Förebyggande insatser för att minska tvångsvård och tvångsåtgärder i psykiatrisk vård av vuxna [Preventive interventions to reduce coercive care and coercive measures in the psychiatric care of adults]. [Internet]. Stockholm: SBU – Statens beredning för medicinsk och social utvärdering; 2019. Available from: https://www.sbu.se/sv/publikationer/sbu-kommentar/forebyggande-insatser-for-att-minska-tvangsvard-och-tvangsatgarder-i-psykiatrisk-vard-av-vuxna/ Accessed 10 Nov 2021.

[5] Bigwood S, Crowe M (2008). ‘It’s part of the job, but it spoils the job’: a phenomenological study of physical restraint. Int J Ment Health Nurs.

[6] Attree M (2001). Patients’ and relatives’ experiences and perspectives of ‘good’ and ‘not so good’ quality care. J Adv Nurs.

[7] Beauchamp TL, Childress JF (2013). Principles of biomedical ethics.

[8] Olofsson B, Gilje F, Jacobsson L, Norberg A (1998). Nurses’ narratives about using coercion in psychiatric care. J Adv Nurs.

[9] Pariseau-Legault P, Vallée-Ouimet S, Goulet MH, Jacob J-D (2019). Systematic Reviews.

[10] Szmukler G, Appelbaum PS (2008). Treatment pressures, leverage, coercion, and compulsion in mental health care. J Ment Health.

[11] Whitecross F, Seeary A, Lee SJ (2013). Measuring the impacts of seclusion on psychiatry inpatients and the effectiveness of a pilot single-session post-seclusion counselling intervention. Int J Ment Health Nurs.

[12] Karger B, Fracasso T, Pfeiffer H (2008). Fatalities related to medical restraint devices—asphyxia is a common finding. Forensic Sci Int.

[13] Krexi L, Georgiou R, Krexi D, Sheppard MN (2016). Sudden cardiac death with stress and restraint: the association with sudden adult death syndrome, cardiomyopathy, and coronary artery disease. Med Sci Law.

[14] Dickson BC, Pollanen MS (2009). Fatal thromboembolic disease: a risk in physically restrained psychiatric patients. J Forensic Legal Med.

[15] Sailas EE, Fenton M. Seclusion and restraint for people with serious mental illnesses. Cochrane Database Syst Rev. 2000. 10.1002/14651858.CD001163. 10.1002/14651858.CD001163 PMC6669266 10796606

[16] Lützén K (1998). Subtle coercion in psychiatric practice. J Psychiatr Ment Health Nurs.

[17] Gustafsson L-K, Wigerblad Å, Lindwall L (2014). Undignified care: violation of patient dignity in involuntary psychiatric care from a nurse’s perspective. Nurs Ethics.

[18] Andersson U, Fathollahi J, Wiklund GL (2020). Nurses’ experiences of informal coercion on adult psychiatric wards. Nurs Ethics.

[19] Olofsson B, Norberg A, Jacobsson L (1995). Nurses’ experience with using force in institutional care of psychiatric patients. Nordic J Psychiatry.

[20] Korkeila H, Koivisto A-M, Paavilainen E, Kylmä J (2016). Psychiatric nurses’ emotional and ethical experiences regarding seclusion and restraint. Ment Health Nurs.

[21] Hem MH, Gjerberg E, Husum TL, Pedersen R (2018). Ethical challenges when using coercion in mental healthcare: a systematic literature review. Nurs Ethics.

[22] Haugom EW, Ruud T, Hynnekleiv T (2019). Ethical challenges of seclusion in psychiatric inpatient wards: a qualitative study of the experiences of Norwegian mental health professionals. BMC Health Serv Res.

[23] Jensen A, Lidell E (2009). The influence of conscience in nursing. Nurs Ethics.

[24] Justesen L, Mik-Meyer N (2011). Kvalitativa metoder.

[25] Burnard P (1991). A method of analyzing interview transcripts in qualitative research. Nurse Educ Today.

[26] Graneheim UH, Lundman B (2004). Qualitative content analysis in nursing research: concepts, procedures, and measures to achieve trustworthiness. Nurse Educ Today.

[27] Vetenskapsrådet. [The Swedish Research Council] God forskningssed [Good Research Practice] [Internet]. Stockholm: Vetenskapsrådet; 2017. Available from: https://www.vr.se/analys/rapporter/vara-rapporter/2017-08-29-god-forskningssed.html. Accessed 3 Sept 2021.

[28] Integritetsskyddsmyndigheten [The Swedish Authority for Privacy Protection]. Dataskyddsförordningen [The General Data Protection Regulation]. [Internet]. Stockholm: Integritetsskyddsmyndigheten; 2016. Available from: https://www.imy.se/verksamhet/dataskydd/det-har-galler-enligt-gdpr/introduktion-till-gdpr/dataskyddsforordningen-i-fulltext/. Accessed 3 Sept 2021.

[29] Etikprövningsmyndigheten [the Swedish ethical review authority]. Vanliga frågor [frequently asked questions]. [Internet]. Stockholm: Integritetsskyddsmyndigheten; 2021. Available from: https://etikprovningsmyndigheten.se/vanliga-fragor/

[30] Kourkouta L, Papathanasiou IV (2014). Communication in nursing practice. Mater Sociomed.

[31] DeWolf Bosek MS (2009). Identifying ethical issues from the perspective of the registered nurse. JONA’s Healthc Law Ethics Regul.

[32] Chieze M, Clavien C, Kaiser S, Hurst S (2021). Coercive measures in psychiatry: a review of ethical arguments. Front Psychiatry.

[33] Pauly B, Varcoe C, Storch J, Newton L (2009). Registred nurses’ perceptions of moral distress and ethical climate. Nurs Ethics.

[34] Eren N (2014). Nurses’ attitudes toward ethical issues in psychiatric inpatient settings. Nurs Ethics.

[35] Paradis-Gagné E, Pariseau-Legault P, Goulet M-H, Jacob JD, Lessard-Deschenes C (2021). Coercion in psychiatric and mental health nursing: a conceptual analysis. Int J Ment Health Nurs.

[36] Aydin Er R, Ersoy N (2017). Ethical problems experienced by nurses who work in psychiatry clinics in Turkey. Journal of Psychiatric Nursing.

[37] Silén M, Ramklint M, Hansson MG, Haglund K (2016). Ethics rounds: an appreciated form of ethics support. Nurs Ethics.

[38] Graneheim UH, Lindgren B, Lundman M (2017). Methodological challenges in qualitative content analysis: A discussion paper. Nurse Educ Today.

[39] Malterud K, Siersma VD, Guassora AD (2016). Sample size in qualitative interview studies: guided by information power. Qual Health Res.

[40] Lagen om etikprövning av forskning som avser människor, 2003:460 [The Act concerning the Ethical Review of Research Involving Humans]. [Internet]. Stockhom: Utbildningsdepartementet [The Ministry of Education and Cultural Affairs]; 2003. Available from: https://www.riksdagen.se/sv/dokument-lagar/dokument/svensk-forfattningssamling/lag-2003460-om-etikprovning-av-forskning-som_sfs-2003-460 .Accessed 16 Jun 2022

